# Efficacy of High-Dose Polyclonal Intravenous Immunoglobulin in COVID-19: A Systematic Review

**DOI:** 10.3390/vaccines10010094

**Published:** 2022-01-09

**Authors:** Daniele Focosi, Massimo Franchini, Marco Tuccori, Mario Cruciani

**Affiliations:** 1 North-Western Tuscany Blood Bank, Pisa University Hospital, 56124 Pisa, Italy; daniele.focosi@gmail.com; 2 Department of Hematology and Transfusion Medicine, Carlo Poma Hospital, 46100 Mantua, Italy; crucianimario@virgilio.it; 3 Division of Pharmacology and Pharmacovigilance, University of Pisa, 56126 Pisa, Italy; marco.tuccori@gmail.com; 4 Unit of Adverse Drug Reaction Monitoring, Pisa University Hospital, 56124 Pisa, Italy

**Keywords:** intravenous immunoglobulins, polyclonal antibodies, immunosuppressants, COVID-19, SARS-CoV-2

## Abstract

Background: Although several therapeutic strategies have been investigated, the optimal treatment approach for patients with coronavirus disease (COVID-19) remains to be elucidated. This systematic review and meta-analysis aimed to evaluate the efficacy and safety of polyclonal intravenous immunoglobulin (IVIG) therapy in COVID-19. Methods: A systematic literature search using appropriate medical subject heading (MeSH) terms was performed through Medline (PubMed), EMBASE, SCOPUS, OVID and Cochrane Library electronic databases. The main outcomes considered were mortality and safety of IVIG versus placebo/standard of care. This review was carried out in accordance with Cochrane methodology including the risk bias assessment and grading of the quality of evidence. Measures of treatment effect were mean differences (MD) together with 95% confidence intervals (CIs) for continuous outcome measures and risk ratio (RR) or MD for binary outcomes. Two reviewers independently extracted data from individual studies, and disagreements were resolved by a third reviewer. Results: A total of 2401 COVID-19 patients from 10 studies (four randomized controlled trials (RCT) and six non-randomized controlled trials (non-RCTs)) were included in the analysis. Participants received IVIG or placebo/standard of care. The use of IVIG was not associated with a significantly reduced risk of death (RR 0.50, 95% CIs 0.18–1.36, *p* = 0.17 for RCTs; RR 0.95, 95% CIs 0.61–1.58, *p* = 0.94 for non-RCTs; low certainty of evidence). IVIG significantly reduced the length of hospital stay (MD −2.24, 95% CIs −3.20/−1.27; *p* = 0.00001; low certainty of evidence), although this difference was significant only for studies evaluating moderate COVID-19 patients. No significant difference was observed in the incidence of overall and serious adverse events between IVIG recipients and controls (very low certainty of evidence). Conclusions: The current evidence from the literature does not support the use of IVIG in COVID-19 patients.

## 1. Introduction

Severe acute respiratory syndrome coronavirus 2 (SARS-CoV-2) caused the coronavirus disease (COVID-19) pandemic beginning in 2019 [1,2]. At the time of writing, more than 4 million people have died from coronavirus disease (COVID-19), and more than 200 million have been infected [3]. Clinicians and researchers have struggled to develop an effective therapeutic protocol to treat and contain the spread of this infectious disease, and more than 300 drugs have been or are being investigated under clinical trials in different parts of the world [4,5]. Among the various therapeutic and prophylactic strategies developed to contain the COVID-19 epidemic, passive immunization by COVID-19 convalescent plasma (CCP) transfusion has been proven effective when CCP was administered early (within 72 h from symptom onset) and with a high titer ( >1:160) of anti-SARS-CoV-2 neutralizing antibodies (nAb) [6,7]. Late disease stages are characterized by an exaggerated immune response, which responds to immunosuppressants: high-dose intravenous immunoglobulin (IVIG), based on previous positive experiences in autoimmune, inflammatory and other infectious diseases, including coronavirus-induced infections [8,9], has been also proposed for COVID-19 [10,11,12,13].

The aim of this review is to systematically analyze the safety and efficacy of the use of high-dose IVIG in patients with COVID-19 (including new primary research) and grade the quality of the available evidence following the Cochrane guidance for methodology.

## 2. Material and Methods

This systematic review was registered at the International Prospective Register of Systematic Reviews (PROSPERO) with the registration number CRD42021281233.

### 2.1. Review Question/Objective

The aim of this systematic review is to evaluate the clinical use of high-dose IVIG for the treatment of COVID-19 patients.

### 2.2. Inclusion and Exclusion Criteria

We included all randomized controlled trials (RCTs) and non-RCTs (i.e., prospective, retrospective, cross-sectional and cohort studies) evaluating the safety and efficacy of high-dose IVIG in patients with COVID-19. We also planned to include controlled non-RCT, considering that only a small number of randomized trials was available. Case reports or case series were excluded from the analysis of this review, as well as studies evaluating hyperimmune IVIG against SARS-CoV-2 or IVIG in viral or other infectious diseases. Non-peer reviewed or ongoing trials were not included in this systematic review, nor were non-comparative studies.

### 2.3. Clinical Setting and Participants

For this systematic review, we considered studies on COVID-19 at any stage of disease severity, from asymptomatic/paucisymptomatic to life-threatening cases. In addition, we included populations of patients with no limitations of age, gender, ethnicity or comorbidities.

### 2.4. Intervention and Outcomes

IVIG treatment at any dose, timing and frequency was evaluated. We planned to include, where available, the following outcomes: all-cause mortality, clinical improvement, serious and non-serious adverse reactions, length of hospital stay and discharge rate, admission to intensive care unit (ICU), length of ICU or hospital stay, need for invasive mechanical ventilation (IMV) and progression to severe disease and adverse events (overall and serious). Severe COVID-19 was defined as the presence of at least one of the following criteria: (1) radiologically confirmed pneumonia; (2) tachypnea with respiratory rate ≥ 30 breaths/min; (3) oxygen saturation (SpO2) ≤ 93% at rest and in room air; and (4) partial pressure of oxygen (PaO2)/fraction of inspired oxygen (FiO2) ≤ 300 mmHg.

### 2.5. Search Methods

For this systematic review we analyzed the medical literature for published articles on the use of IVIG in COVID-19 patients. A literature search of the MEDLINE (through PUBMED), EMBASE, SCOPUS, OVID and Cochrane Library electronic databases was carried out from 1 January 2020 to 31 August 2021, using English language as a restriction. Only articles published following a peer-reviewing process were included in the final analysis. The medical subject heading (MeSH) and key words used were: (“COVID-19” OR “SARS-CoV-2” OR “coronavirus disease 2019”) AND (“IVIG” OR “immunoglobulin”). We also screened the reference lists of the most relevant review articles for additional studies not captured in our initial literature search.

### 2.6. Study Selection and Data Extraction

All titles were screened by two independent reviewers (MC and MF). Eligibility assessment was based on the title or abstract and on the full text if necessary. Full texts of possibly eligible articles were evaluated independently by two reviewers (MC and MF). Studies were selected independently by two reviewers (MF and MC) with disagreements resolved by a third reviewer (DF). The two reviewers also independently extracted quantitative and qualitative data from each selected study (Table 1). Quantitative tabulation of results includes: first author name and year of publication, the type of the study, the disease severity, the population size (intervention and control groups), treatment (intervention and control groups), adverse reactions and main results.

### 2.7. Assessment of the Methodological Quality of Published Clinical Studies

Two review authors (MF, MC) independently assessed the risk of bias of each included study following the domain-based evaluation described in the *Cochrane Handbook for Systematic Reviews of Interventions* [14]. They discussed any discrepancies and achieved consensus on the final assessment. 

We considered both RCTs and controlled non-RCT. Within-trial risk of bias was assessed, using the Cochrane ROB tool for RCTs and the ROBINS-I tool for non-RCTs. The Cochrane ‘Risk of bias’ tool for RCTs addresses six specific domains: sequence generation, allocation concealment, blinding, incomplete data, selective outcome reporting and other issues relating to bias. The methodological quality of observational studies was assessed with the ROBINS-1 tool [15,16,17]. This tool includes seven specific bias domains, pre-intervention and post-intervention, and it has to be performed as an outcome-based assessment for each outcome reported for a trial. The domains are: (1) confounding; (2) selection of participants; (3) classification of intervention; (4) deviation from interventions (biases that arise when there are systematic differences between the care provided to experimental intervention and comparator groups, beyond the assigned interventions); (5) missing outcome; (6) measurement of outcomes (blinding of outcome assessors aims to prevent systematic differences in measurements between intervention groups, but it is less common in non-RCTs than in RCTs; and (7) selection of reported result overall. 

**Figure 1:**
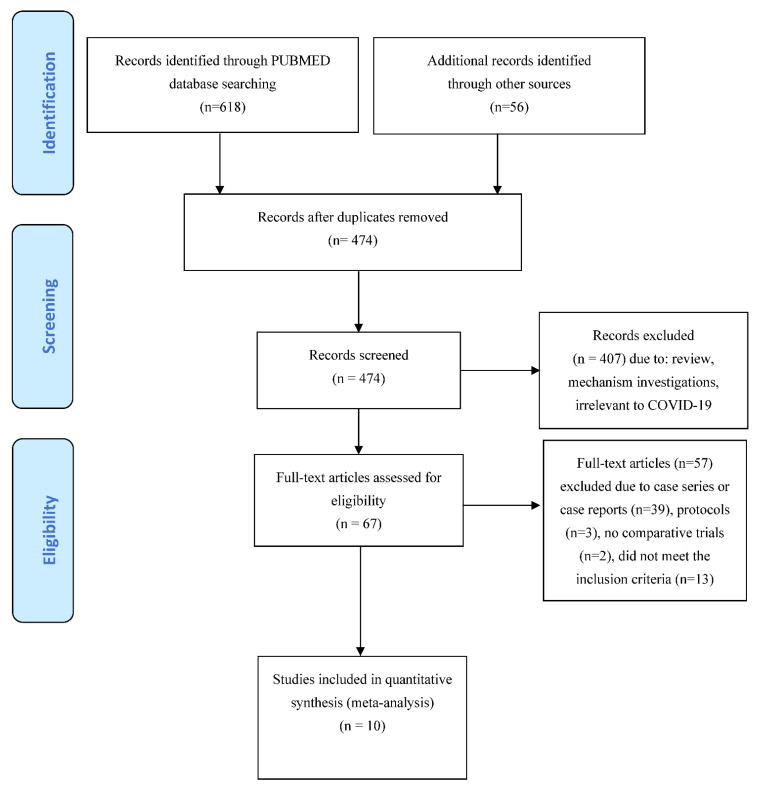
PRISMA flow diagram of study selection.

For both RCTs and non-RCTs we have presented our assessment of risk of bias using two ‘Risk of bias’ summary figures: (1) a summary of bias for each item across all studies; and (2) a cross-tabulation of each trial by all the ‘Risk of bias’ items.

### 2.8. Effect of Intervention

Measures of treatment effect were mean differences (MD) together with 95% confidence intervals (CIs) for continuous outcome measures and risk ratio (RR) or MD for binary outcomes. When necessary, disagreement was resolved by consensus and by a third reviewer (DF). For continuous measures, the score had to be reported as mean and standard deviation (SD); when reports provided medians and interquartile range, pooling of data was not performed.

The study weight was calculated using the Mantel–Haenszel method. We assessed statistical heterogeneity using t^2^, Cochran’s Q and *I*^2^ statistics [18]. The *I*^2^ statistic describes the percentage of total variation across trials due to heterogeneity rather than sampling error. In the case of no heterogeneity (*I*^2^ = 0), studies were pooled using a fixed-effects model. When values of *I*^2^ were >0, a random-effects analysis was undertaken.

### 2.9. Subgroup Analyses

We anticipated heterogeneity in the design and reporting of studies and, to deal with heterogeneity, we planned to carry out subgroup analyses of the outcomes “mortality” and “length of hospital stay” in treatment control groups according to baseline clinical conditions (e.g., moderate vs. severe COVID-19).

### 2.10. ‘Summary of Findings’ Tables

We used the principles of the GRADE system to assess the quality of the body of evidence associated with specific outcomes and constructed ’Summary of findings’ tables using REVMAN 5.4 (The Cochrane Collaboration, London, UK) [19,20]. These tables present key information concerning the certainty of the evidence, the magnitude of the effects of the interventions examined and the sum of available data for the main outcomes. The ’Summary of findings’ tables also include an overall grading of the evidence related to each of the main outcomes using the GRADE approach, which defines the certainty of a body of evidence as the extent to which one can be confident that an estimate of effect or association is close to the true quantity of specific interest. The certainty of a body of evidence involves consideration of within-trial risk of bias (methodological quality), directness of evidence, heterogeneity, precision of effect estimates, and risk of publication bias. We have presented the following outcomes in the ‘Summary of findings’ table: (i) mortality; (ii) length of hospital stay and (iii) adverse events. All calculations were conducted using REVMAN 5.4.

## 3. Results

Electronic and manual searches yielded 674 potentially relevant studies (Figure 1). Six hundred and seven articles were excluded after preliminary screening. The remaining 67 articles were deemed potentially eligible, and their full text was assessed. Fifty-five articles were then excluded because of case series or case reports, protocols or because they did not meet the inclusion criteria. Two additional studies were excluded because, although they focused on IVIG use in COVID-19 patients, they were not controlled trials [21,22]. Thus, for this systematic review we considered 10 studies fulfilling our pre-specified criteria, including 2401 COVID-19 patients [23,24,25,26,27,28,29,30,31,32]. Four studies were RCT [25,29,30,32], and six studies were non-RCT (all retrospective) [24,26,27,28,31,32]. Five studies were conducted in China, two in Iran and one each in India, the USA and Turkey. In two studies, IVIG use was restricted to non-severe COVID-19 cases [27,29], while in the other eight trials IVIGs were used in severe or critical patients. Table 1 summarizes the main characteristics and results of these studies. The dosage and timing of IVIG administration was greatly variable between studies. The comparator group included standard therapy (nine studies) or placebo (one study) [25].

**Table 1:** Characteristics of the studies included.

First Author, Year [Ref.]	Type of Study	Disease Severity	Population Size (Intervention/Control)	Single IVIG Dose per Day (Intervention/Control)	Duration (Days)	Cumulative Dose	Control	Safety	Main Results
Cao, 2021 [23]	RCS	Severe COVID-19	26/89	0.4–1 g/kg	2–5 days	2 g/kg	ST	No AEs	High-dose IVIG reduced 28-day mortality (HR 0.24, 95% CI 0.06–0.99; *p* < 0.001). Early treatment (within 7 days of onset) was associated with greater benefit
Esen, 2021 [24]	RCS	Severe COVID-19	51/42	0.4 g/kg *	5 days	2 g/kg	ST	NR	IVIG significantly prolonged median survival time (68 versus 18 days, *p* = 0.014)
Gharebaghi, 2020 [25]	RCT	Severe COVID-19	30/29	0.3 g/kg *	3 days	0.9 g/kg	placebo	NR	IVIG significantly reduced mortality rate (aOR 0.003, 95% CI 0.001–0.815; *p* = 0.042)
Hou, 2021 [26]	RCS	Severe COVID-19	47/66	0.5 g/kg	NR	NR	ST	NR	IVIG did not improve in-hospital mortality rates or the need for mechanical ventilation
Huang, 2021 [27]	RCS	Non-severe COVID-19	45/594	0.13 g/kg (8 patients) *	3 days	0.5 g/kg	ST	NR	No benefit was observed with IVIG in terms of mortality rate, progression to severe disease or length of hospital stay
				0.13 g/kg (13 patients) *	5 days	0.7 g/kg			
				0.26 g/kg (16 patients) *	3 days	0.8 g/kg			
				0.26 g/kg (8 patients) *	5 days	1.3 g/kg			
Liu, 2021 [28]	RCS	Severe COVID-19	421/429	0.13 g/day	9.5 days	1.3 g/kg	ST	NR	IVIG was not associated with significant changes in 28-day mortality in severe COVID-19 patients
Raman, 2021 [29]	RCT	Non-severe COVID-19	50/50	0.4 g/kg	5 days	2 g/kg	ST	17 (34%) mild to moderate	Duration of hospital stay was significantly lower in IVIG group (7.7 vs. 17.5 days, *p* = 0.0001)
Sakoulas, 2020 [30]	RCT	Severe COVID-19	16/17	0.5 g/kg	3 days	1.5 g/kg	ST	No AEs	IVIG improved hypoxia and reduced hospital length of stay and progression to mechanical ventilation
Shao, 2020 [31]	RCS	Severe or critical COVID-19	174/151	0.1 g/kg (100 patients) 0.5 g/kg (74 patients)	5–15 days (not specified according to daily dose)	0.5–5 g/kg (not specified according to daily dose)	ST	NR	Early administration (≤7 days after hospital admission) with high dose (>15 g/day) of IVIG significantly reduced 60-day mortality
Tabarsi, 2021 [32]	RCT	Severe COVID-19	52/32	0.4 g/kg	3 days	1.2 g/kg	ST	NR	No benefit was observed with IVIG in terms of mortality rate and need for mechanical ventilation

Abbreviations: AE, adverse events; aOR, adjusted odds ratio; BW, body weight; CI, confidence intervals; IVIG, intravenous immunoglobulin; HR, hazard ratio; NR, not reported; RCS, retrospective cohort study; RCT, randomized controlled trial; ST, standard therapy. * Assuming a mean recipient body weight of 75 kg.

### 3.1. Risk of Bias in Included Studies

Of the four RCTs, three were at high risk of performance (open-label trials), three were at unclear risk of detection bias (because it was unclear whether the assessors were blinded to treatment allocation) and two at unclear risk of selection bias (because no clear information on allocation concealment were provided) (Supplementary File S1). According to the ROBINS-I tool, for the outcome mortality, four non-RCTs were judged to be at high risk of confounding bias, and two at unclear risk (Supplementary File S1). For selection bias, three non-RCTs were judged to be at high risk, and one at unclear risk. All the non-RCTs were judged to be at unclear risk of bias for the domain bias in measurement “classification of interventions” due to the retrospective nature of these studies.

### 3.2. Effects of Interventions

**Figure 2:**
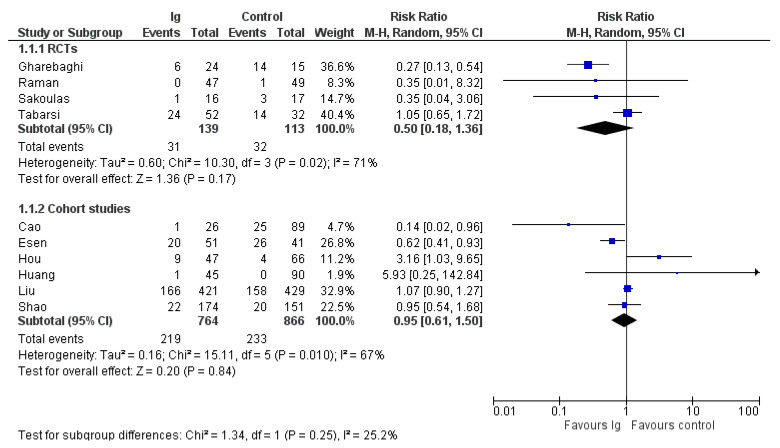
Forest plot of mortality.

**Figure 3:**
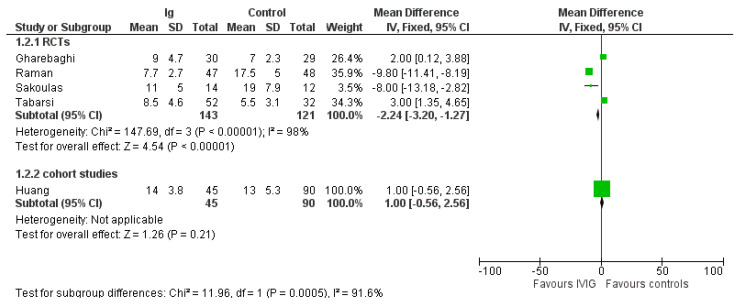
Forest plot of length of hospital stay.

A summary of the outcomes reported in the included study is provided in Table 1 and the Supplementary File S1 (Data and Analyses). The most commonly reported outcomes were mortality and length of hospital stay (Figure 2 and Figure 3). For the other outcomes (see the Supplementary File S1), it was not possible to perform a pooled analysis because they were inconsistently reported or because, for continuous variables, values were reported as median and interquartile range.

### 3.3. Mortality

Data on mortality were reported in all 10 trials included in the review. In the four RCTs, there were 31 deaths out of 139 patients in the IVIG group compared to 32/113 in the control group (risk ratio (RR), 0.50; 95% CIs, 0.18/1.36; *p* = 0.17) (Figure 2); in the six non RCTs, there were 219 deaths out of 764 patients in IVIG group compared to 233/866 in the control group (RR, 0.95; 95% CIs, 0.61/1.58; *p* = 0.94). In both cases the level of certainty was low due to imprecision and inconsistency. The subgroup analysis (see Supplementary File S2) according to the baseline conditions did not show differences in mortality rates in studies conducted in patients with moderate COVID-19 and in patients with severe/critical illness, both in RCTs (low quality of evidence) or in non-RCTs (very low quality of evidence) (Table 2).

### 3.4. Length of Hospital Stay

In the four RCTs IVIG reduced length of hospital stay compared to controls (MD, −2.24 days; 95% CIs, −3.20/−1.27; *p* = 0.00001) (Figure 3). Low certainty of evidence was due to inconsistency and ROB. The effect was driven mostly by inclusion of patients with a moderate COVID-19 infection. Indeed, in the two studies enrolling severe patients (see Supplementary File S2), the difference in length of hospital stay favored controls compared to IVIG (MD, 2.57 days; 95% CIs, 1.33/3.80; *p* < 0.0001; low quality of evidence), while in studies evaluating moderate patients, the difference favored IVIG compared to controls (RR, −9.64; 95% CIs, −11.18/−8.1; *p* < 0.00001; low quality of certainty) (Table 2). Only one non-RCT [27] was evaluated for length of hospital stay.

Pooled analyses of RCTs and non-RCTs for the outcomes mortality and length of hospital stay did not significantly affect the effect size of the intervention, and the heterogeneity in the overall analyses remained high (data not shown).

### 3.5. Adverse Events

**Figure 4:**
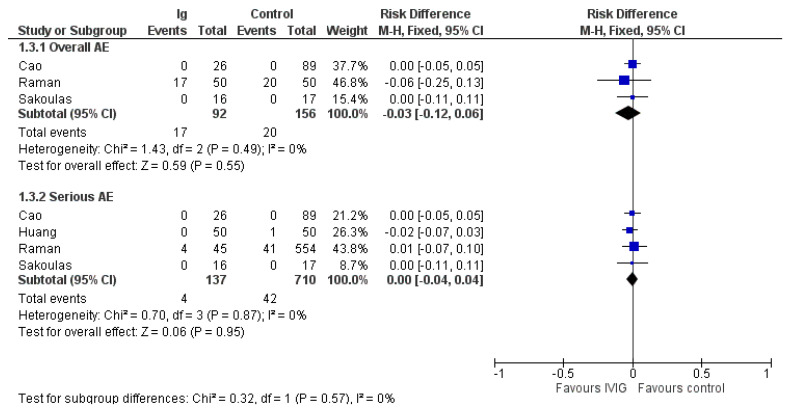
Forest plot of adverse events.

A total of 6 out of 10 studies did not mention adverse events at all. Overall adverse events were reported in three trials [23,29,30] and serious adverse events in four trials [23,27,29,30] (Figure 4). Overall adverse events were reported in 17/92 patients receiving IVIG and 20/156 patients receiving standard therapy (risk difference (RD) −0.03; 95% CIs, −0.12/0.06; very low quality of evidence due to imprecision and serious risk of bias). Serious adverse events were rarely reported either in IVIG recipients (4/137) or controls (42/710) (RD, 0.00; 95% CIs, −0.04/0.04; very low quality of evidence due to imprecision and serious risk of bias (Table 2).

## 4. Discussion

Following the first trial demonstrating the efficacy of high-dose IVIG in patients with severe COVID-19 [23], other studies were conducted in this setting to assess the impact of IVIG on various clinical outcomes, mainly on mortality. High-dose polyclonal IVIGs are supposed to be immunosuppressive at doses in the range of 2 g per kg of recipient body weight. Given that COVID-19 largely resembles an autoimmune disorder in the late stages, IVIGs are presumed to act as an immune modulator by dampening the excessive immune response to SARS-CoV-2 which drives pathology [33,34]. A systematic review and meta-analysis by Xiang and colleagues on three clinical trials and three cohort studies [12] found that the effect of IVIG was associated with the severity of COVID-19, as it was highest in the critical subgroup. Our updated systematic review, performed on a higher number of studies and including more patients, gave different results. Although the length of hospitalization was significantly reduced in the IVIG group versus controls, a subgroup analysis showed that this effect was present only in patients with moderate disease. Regarding the main outcome “mortality”, the use of IVIG was not associated with a significantly reduced risk of death, independently of COVID-19 severity or type of study (RCT or non-RCT). Such results are in accordance with a recently published living systematic review and network meta-analysis [35].

The majority of trials included in this systematic review were judged to be at high risk or at unclear risk of bias in many of the items of the ROB tool for RCTs and the ROBIN-1 tool for non-RCTs. Of the four RCTs, three were at high risk of performance (open-label trials), three were at unclear risk of detection bias (because it was unclear if the assessor was blinded to treatment allocation) and two at unclear risk of selection bias (because no clear information on allocation concealment was provided). Of the six non-RCTs, four were judged at high risk and two at unclear risk of confounding bias; three were judged at high risk and one at unclear risk of selection bias; all the non-RCTs were judged at unclear risk of bias for the domain bias in the measurement classification of interventions due to the retrospective nature of these studies. Moreover, considering the observed heterogeneity and imprecision in the effect size of the outcomes analyzed, the level of certainty of the available evidence was graded as low or very low. Another potential bias of this systematic review was the wide heterogeneity of IVIG doses in different studies, ranging from 0.1 to 1 g/kg/day. It is therefore possible that sub-immunosuppressive doses have diluted the beneficial effect of fully immunosuppressive doses in the meta-analysis.

Adverse events were reported inconsistently and only in 4 of the 10 selected trials. Although overall adverse events and serious adverse events were rarely reported either in IVIG recipients or controls, the available evidence was graded as very low level of certainty due to inconsistency (small number of events and participants) and serious risk of bias.

In conclusion, although IVIGs appeared to be safe in COVID-19 patients, their use did not result in a significant reduction in mortality. Thus, the results of this systematic review and meta-analysis do not support the use of high-dose IVIG in COVID-19 patients.

Despite the initial signs of heterologous immunity to SARS-CoV-2 from previous seasonal coronavirus infection [36,37], pre-pandemic sera have been shown to be devoid of neutralizing activity [38], making the occurrence of neutralizing activity extremely unlikely in lots manufactured from plasma collected before 2021. However, the situation is rapidly evolving, with most plasma donors worldwide becoming seropositive because of convalescence and/or vaccination. Baxter reported seropositivity in lots released since September 2020. From there, values steadily increased, in correlation with the cumulative COVID-19 incidence, to reach a mean of 36.7 IU/mL, and 93% of lots were positive by January 2021. Extrapolating the correlation, the authors estimated that IVIGs could have reached an anti-SARS-CoV-2 potency of ~400 IU/mL (i.e., a dose similar to that contained in a CCP unit) by July 2021. Therefore, further trials evaluating the last batches of IVIG are needed to assess whether the addition of anti-SARS-CoV-2 nAbs to their immunomodulatory effects will translate into a higher clinical efficacy.

## Figures and Tables

**Table 2:** Summary of findings table.

Immunoglobulin Compared with Standard Treatment for COVID-19						
Patient or Population: Adults with COVID-19 Settings: Both Outpatients and Hospitalized pts Intervention: IVIG Comparison: Standard Treatment						
Outcomes	Illustrative Comparative Risks * (95% CI)		Relative Effect (95% CI)	No. of Participants (Studies)	Quality of the Evidence (GRADE)	Comments
	Assumed Risk	Corresponding Risk				
	Control	IVIG				
Mortality—RCTs (28 days)	Overall population with COVID-19		RR 0.50 (0.18/1.36) RR 0.35 (0.06/2.10) RR 0.54 (0.14/2.09)	252 (4) 125 (2) 111 (2)	⊕⊕⊝⊝ low ^1^	It is unclear whether IVIG reduces mortality compared to standard treatment in the overall populations of pts with COVID-19 or in moderate or severe COVID-19 pts
	Mean mortality was 28.3%	14.5% (5.0/38.4%)				
	Low-risk population (pts with moderate disease)					
	Mean mortality was 6.0%	2.1% (0.3/12.54%)				
	High-risk population (pts with severe/critical disease)					
	Mean mortality was 59.5%	32.1% (8.3/124.3%)				
Mortality—Cohort studies	Overall population with COVID-19		RR 0.95 (0.61/1.50)	6 (1630)	⊕⊕⊝⊝ low ^2^	It is unclear whether IVIG reduces mortality compared to standard treatment in COVID-19. The differences were not significant in subgroup analyses of pts with moderate or severe disease either.
	Mean mortality was 26.9%	25.2% (16.4/40.3%)				
Length of Hospital stay (days)	The mean hospital stay is 12.25	10.1 (9.05/10.98)	RD−2.24 (−3.20/−1.27)	4 (264)	⊕⊕⊝⊝ low ^2^	IVIG reduces LHS compared to standard treatment. The effect was driven mostly by inclusion of pts with moderate COVID-19 infections. Indeed, in the 2 studies enrolling severe pts (see Supplementary File S2), the difference in LHS favored controls compared to IVIG (RD, 2.57; 95% CIs, 1.33/3.80; *p* < 0.0001; low quality of evidence), while in studies evaluating moderate pts, the difference favored IVIG compared to controls.(RR, −9.64; 95% Cis, −11.18/−8.1; *p* < 0.00001; low quality of certainty)
Adverse events - Overall AE	The mean occurrence of AE was 12.8%	12.5% (11.6/13.4%)	RD −0.03 (−0.12/0.06)	3 (248)	⊕⊝⊝⊝ very-low ^3^	Mean occurrence of AE was similar in IVIG recipients and controls
- Serious AE	The mean occurrence of serious AE was 5.9%	5.9% (5.5/6.3%)	RR 0.00 (−0.04/0.04)	4 (848)	⊕⊝⊝⊝ very-low ^3^	Mean occurrence of serious AE was similar in IVIG recipients and controls

* The basis for the assumed risk is the mean control group risk across studies. The corresponding risk (and its 95% confidence interval) was based on the assumed risk in the comparison group and the relative effect of the intervention (and its 95% CI). CI: Confidence interval; RR: Risk Ratio; RD, risk difference; AE, adverse events; IVIG, intravenous iommunoglobulin. GRADE Working Group grades of evidence. High quality: Further research is very unlikely to change our confidence in the estimate of effect. Moderate quality: Further research is likely to have an important impact on our confidence in the estimate of effect and may change the estimate. Low quality: Further research is very likely to have an important impact on our confidence in the estimate of effect and is likely to change the estimate. Very low quality: We are very uncertain about the estimate. ^1^ Downgraded for imprecision and inconsistency. ^2^ Downgraded for inconsistency and ROB. ^3^ Downgraded for imprecision and twice downgraded for serious ROB.

## Supplementary Materials

The following are available online at https://www.mdpi.com/article/10.3390/vaccines10010094/s1, Supplementary File S1: Risk of bias tables. Supplementary File S2: Risk of bias graphs and forest plots of comparison.

## Abbreviations

IVIG: intravenous immunoglobulins; COVID-19: coronavirus disease 2019; SARS-CoV-2: severe acute respiratory syndrome coronavirus 2; CCP: COVID-19 convalescent plasma; HS: hyperimmune serum.
