# Supplementary material for: Efficacy of High-Dose Polyclonal Intravenous Immunoglobulin in COVID-19: A Systematic Review
Source: Vaccines (Basel). 2022 Jan 9;10(1):94. doi: 10.3390/vaccines10010094 (PMC8779789; doi:10.3390/vaccines10010094)
Supplement: Supplementary file 1 [file vaccines-10-00094-s001.zip › Supplementary figures.pdf]

Supplementary figures

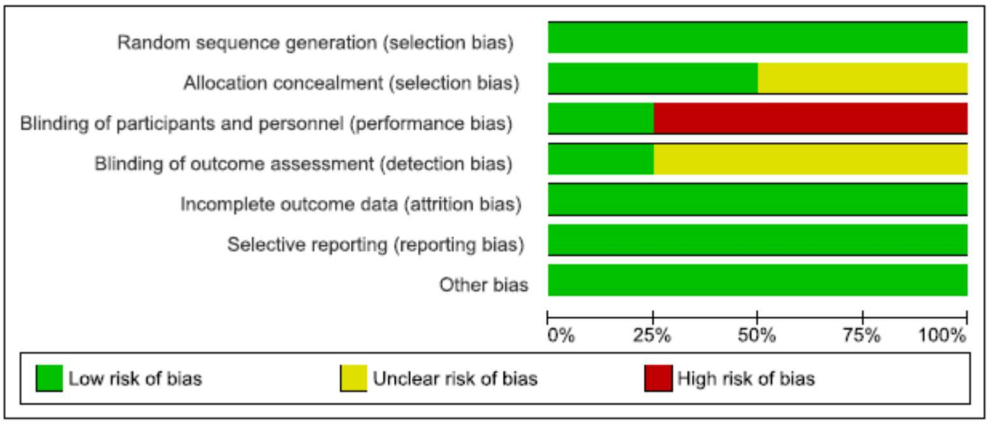

Risk of bias graph: review authors' judgements about each risk of bias item presented as percentages across all included RCTs.

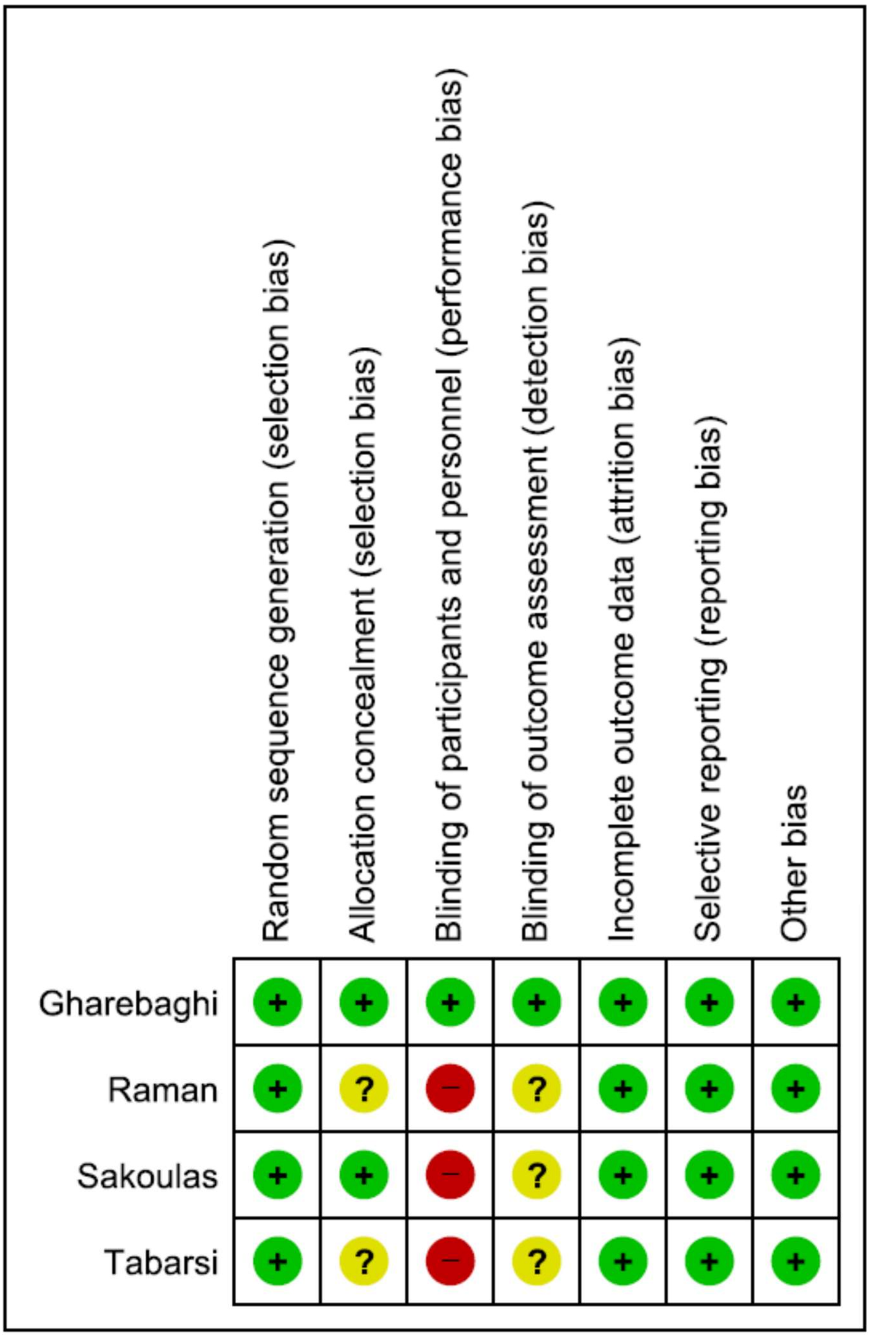

Risk of bias summary: review authors' judgements about each risk of bias item for each included RCTs.

Supplementary figures

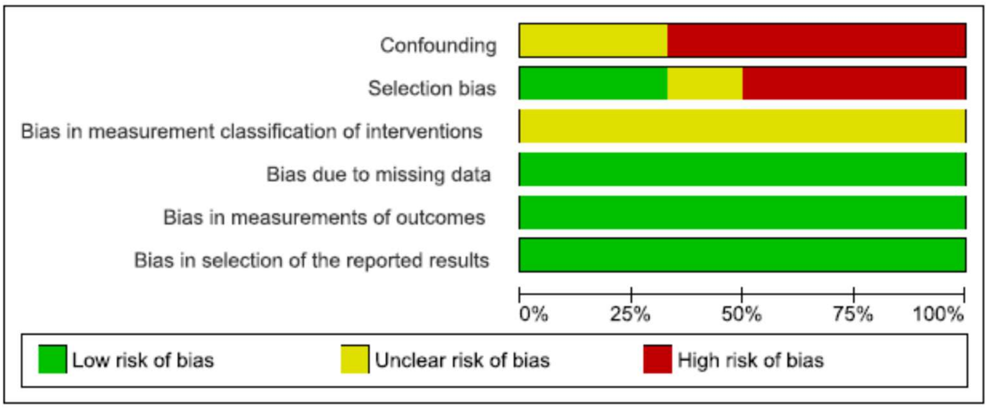

Risk of bias graph: review authors' judgements about each risk of bias item presented as percentages across all included non-RCTs.

Supplementary figures

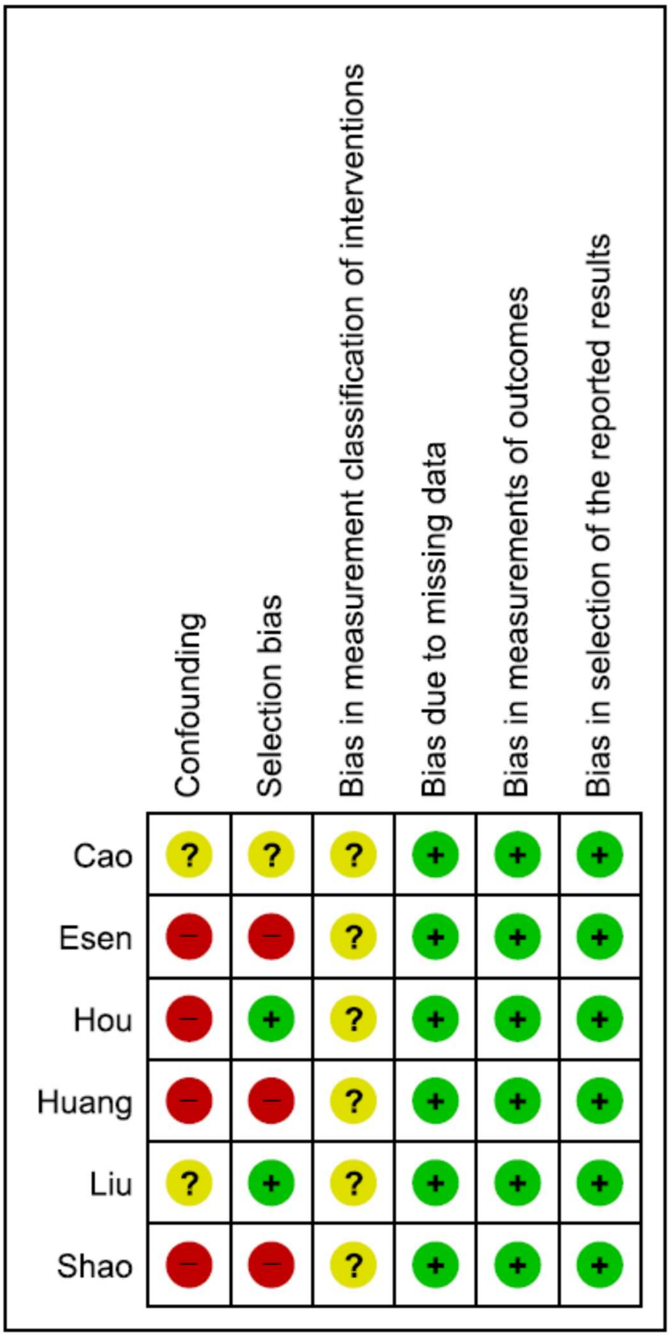

Risk of bias summary: review authors' judgements about each risk of bias item for each included non-RCTs.

Supplementary figures

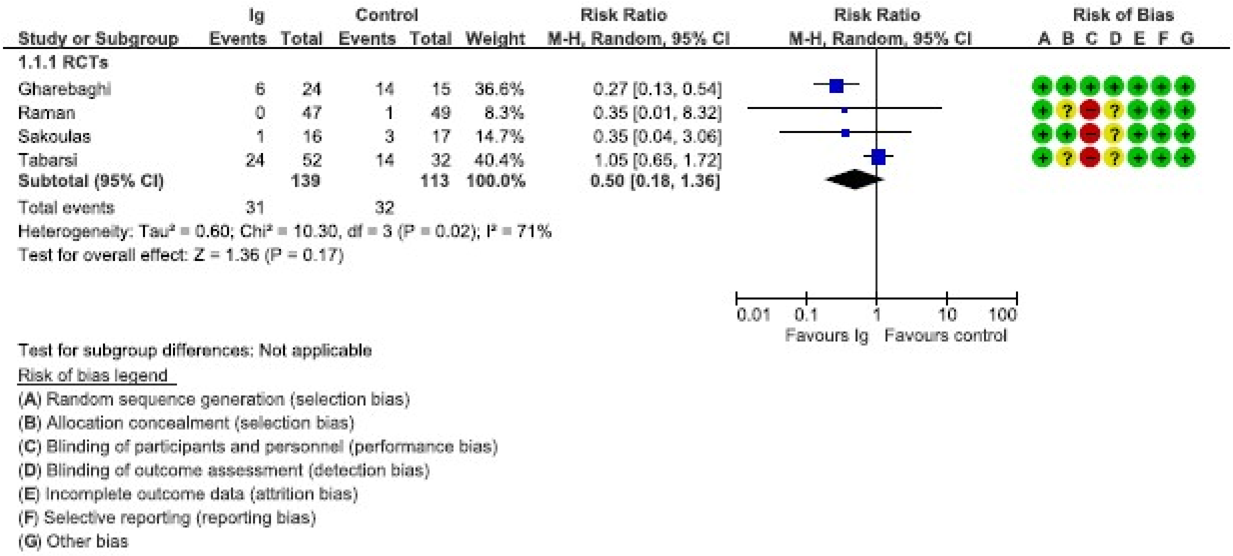

Forest plot of comparison. Outcome: Mortality. Overall data (moderate and severe COVID-19) from the 4 RCTs. IVIG did not decrease 28-days mortality compared to controls. Low level of certainty; downgraded for inconsistency (due to heterogeneity) and imprecision (95% CI includes line of no effect); although some of the included studies were at high risk of performance bias, we did not downgrade the available evidence for ROB since masking has limited importance for the outcome of mortality compared to subjective outcomes

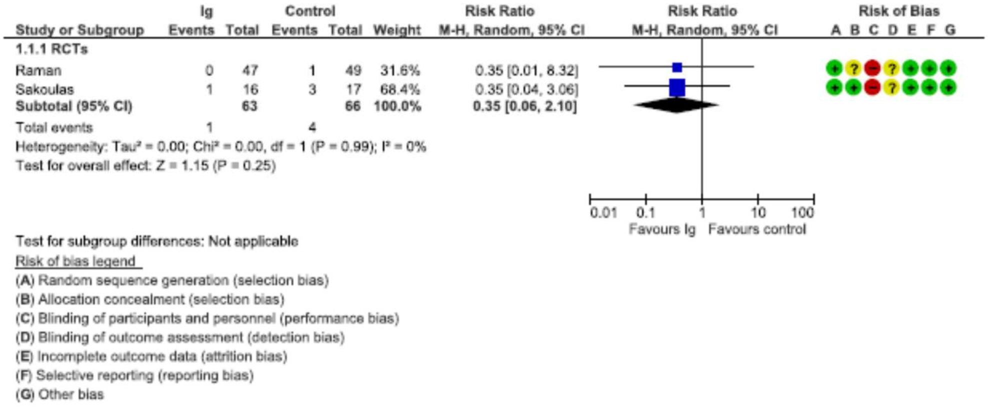

Forest plot of comparison. Outcome: Mortality. Data from the 2 RCTs evaluating moderate COVID-19. In subgroup analysis of studies evaluating moderate COVID-19 cases (Raman and Sakoulas), IVIG did not decrease 28-days mortality compared to controls (low level of certainty, downgraded for inconsistency and imprecision).

Supplementary figures

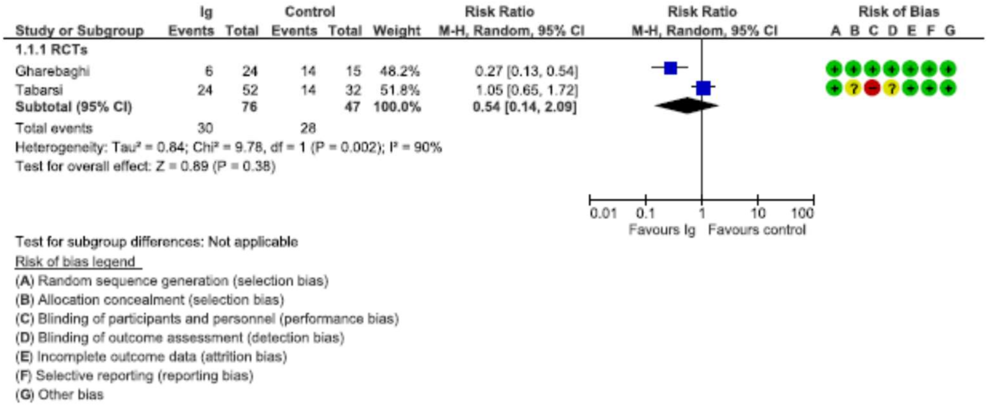

Forest plot of comparison. Outcome: Mortality. Data from the 2 RCTs evaluating severe COVID-19. In subgroup analysis of studies including severe COVID-19 cases (Gharabaghi e Tabarsi), IVIG did not decrease 28-days mortality compared to controls (low level of certainty, downgraded for inconsistency and imprecision).

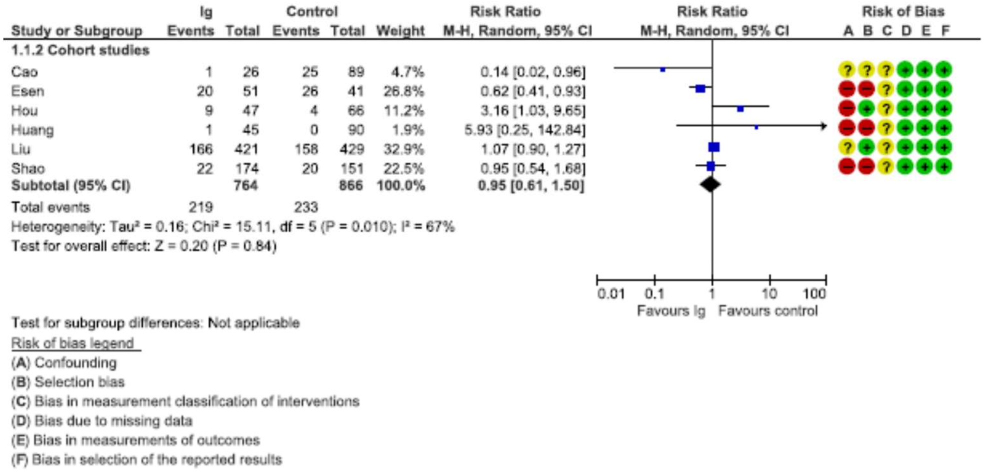

Forest plot of comparison. Outcome: mortality in cohort studies. Most studies included severe COVID-19 pts. Exclusion of moderate case (in the study of Huang, and in subset of pts in the Shao study) did not affect the effect size compared to the overall analysis. Very- low quality of certainty; downgraded for imprecision, inconsistency, and ROB (confounding and selection bias).

Supplementary figures

Study or Subgroup	Ig Mean	Ig SD	Ig Total	Control Mean	Control SD	Control Total	Weight	Mean Difference IV, Fixed, 95% CI
**1.2.1 RCTs**								
Gharebaghi	9	4.7	30	7	2.3	29	43.5%	2.00 [0.12, 3.88]
Tabarsi	8.5	4.6	52	5.5	3.1	32	56.5%	3.00 [1.35, 4.65]
**Subtotal (95% CI)**			**82**			**61**	**100.0%**	**2.57 [1.33, 3.80]**
Heterogeneity: Chi² = 0.62, df = 1 (P = 0.43); I² = 0%								
Test for overall effect: Z = 4.06 (P < 0.0001)								

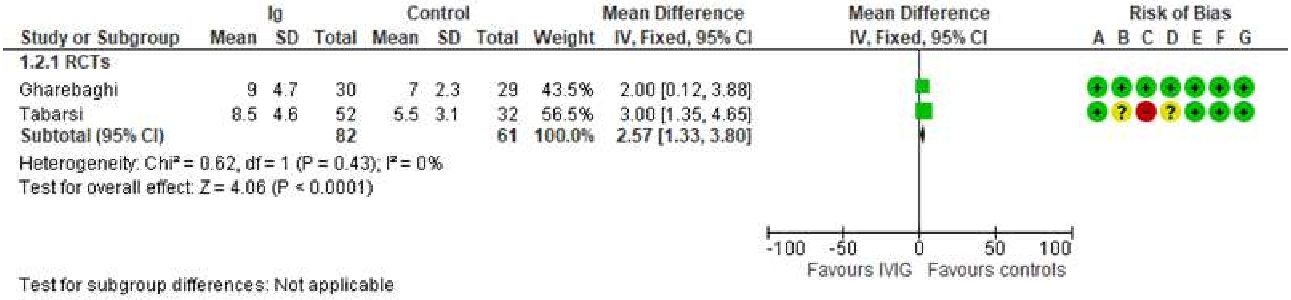

Test for subgroup differences: Not applicable

Risk of bias legend
(A) Random sequence generation (selection bias)
(B) Allocation concealment (selection bias)
(C) Blinding of participants and personnel (performance bias)
(D) Blinding of outcome assessment (detection bias)
(E) Incomplete outcome data (attrition bias)
(F) Selective reporting (reporting bias)
(G) Other bias

Forest plot of comparison. Outcome: Length of hospital stay. Subgroup analysis of trials including severe COVID-19 pts. The difference in LHS favoured controls compared to IVIG; low quality of evidence (downgraded for imprecision and ROB).

Study or Subgroup	Ig Mean	Ig SD	Ig Total	Control Mean	Control SD	Control Total	Weight	Mean Difference IV, Fixed, 95% CI
**1.2.1 RCTs**								
Raman	7.7	2.7	47	17.5	5	48	91.2%	-9.80 [-11.41, -8.19]
Sakoulas	11	5	14	19	7.9	12	8.8%	-8.00 [-13.18, -2.82]
**Subtotal (95% CI)**			**61**			**60**	**100.0%**	**-9.64 [-11.18, -8.10]**
Heterogeneity: Chi² = 0.42, df = 1 (P = 0.52); I² = 0%								
Test for overall effect: Z = 12.28 (P < 0.00001)								

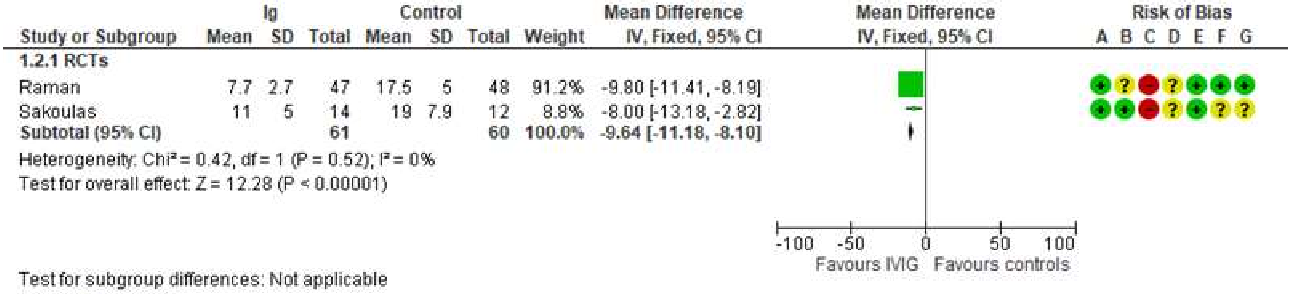

Test for subgroup differences: Not applicable

Risk of bias legend
(A) Random sequence generation (selection bias)
(B) Allocation concealment (selection bias)
(C) Blinding of participants and personnel (performance bias)
(D) Blinding of outcome assessment (detection bias)
(E) Incomplete outcome data (attrition bias)
(F) Selective reporting (reporting bias)
(G) Other bias

Forest plot of comparison. Outcome: Length of hospital stay. Subgroup analysis of trials including moderate COVID-19 pts. The difference in LHS favoured IVIG compared to controls; low quality of evidence (downgraded for imprecision and ROB).
