# Supplementary material for: Efficacy of High-Dose Polyclonal Intravenous Immunoglobulin in COVID-19: A Systematic Review
Source: Vaccines (Basel). 2022 Jan 9;10(1):94. doi: 10.3390/vaccines10010094 (PMC8779789; doi:10.3390/vaccines10010094)
Supplement: Supplementary file 1 [file vaccines-10-00094-s001.zip › Supplementary tables.pdf]

Data and analyses

1 Ig vs controls

Outcome or Subgroup	Studies	Participants	Statistical Method	Effect Estimate
1.1 Mortality	10		Risk Ratio (M-H, Random, 95% CI)	Subtotals only
1.1.1 RCTs	4	252	Risk Ratio (M-H, Random, 95% CI)	0.50 [0.18, 1.36]
1.1.2 Cohort studies	6	1630	Risk Ratio (M-H, Random, 95% CI)	0.95 [0.61, 1.50]

MAIN CHARACTERISTICS OF RANDOMIZED STUDIES, INCLUDING RISK OF BIAS

Characteristics of included studies

Gharebaghi

**Methods**	a randomized placebo-controlled double-blind clinical trial.
**Participants**	Fifty-nine patients with severe COVID-19 infection who did not respond to initial treatments were randomly assigned into two groups.
**Interventions**	One group received IVIg (human)—four vials daily for 3 days (in addition to initial treatment), while the other group received a placebo.
**Outcomes**	in-hospital mortality
**Notes**	Inadequate response to initial treatment was defined as the lack of improvement of dyspnea, fever, and hypoxemia (satO2 less than 90%), as well as the need for oxygenation to maintain satO2 above 90% after 48 h of commencing treatment

Risk of bias table

**Bias**	**Authors' judgement**	**Support for judgement**
Random sequence generation (selection bias)	Low risk	using a computer-generated randomization schedule
Allocation concealment (selection bias)	Low risk	The only individual that was aware of the treatment was the pharmacist of the study center.
Blinding of participants and personnel (performance bias)	Low risk	Neither patients nor physicians nor data analysts were aware of treatment versus placebo membership. The only individual that did was the pharmacist of the study center. Placebo and IVIg vials were similar in appearance and contained a similar volume of solution. Placebo vials contained saline solution.
Blinding of outcome assessment (detection bias)	Low risk	see above
Incomplete outcome data (attrition bias)	Low risk	no risk of attrition bias detected
Selective reporting (reporting bias)	Low risk	all outcomes reported
Other bias	Low risk	None noted

Raman

**Methods**	An open-label, multicenter, comparative, randomized study
**Participants**	COVID-19 patients with moderate pneumonia.
**Interventions**	One hundred eligible patients were randomized in 1:1 ratio either to receive IVIG + standard of care (SOC) or SOC.
**Outcomes**	The primary endpoint was number of days from initiation of treatment to hospital discharge. The secondary endpoints were as follows: time taken for improvement of clinical parameters, which included number of days for normalization of body temperature (94% on room air), and duration of cough; duration of mechanical ventilation from day 0 to 28; number of deaths during the follow-up of 28 days; and proportion of patients with negative RT-PCR during the study period on day 14, on day 28, or end of the study period
**Notes**	Patients with moderate pneumonia were defined as follows: body temperature ≥38.0°C or PaO2 /FiO2 100–300 mmHg or respiratory rate >24/minutes and oxygen saturation 90%–93% on room air or lung involvement confirmed with chest x-rays.

Risk of bias table

Bias	Authors' judgement	Support for judgement
Random sequence generation (selection bias)	Low risk	The biostatistician generated random numbers using block randomization with block sizes of 4 using SAS program and allocated eligible patients to either Ig or control
Allocation concealment (selection bias)	Unclear risk	see above
Blinding of participants and personnel (performance bias)	High risk	open label
Blinding of outcome assessment (detection bias)	Unclear risk	no information provided
Incomplete outcome data (attrition bias)	Low risk	Intent-to-treat population consisted of 100 patients who received either 1 dose of study drug (IVIG) or SOC as stipulated in the protocol.4 patients discontinued from the study: 1 patient was lost to follow-up, and 3 patients had treatment interruption and study withdrawal because of adverse events.
Selective reporting (reporting bias)	Low risk	all outcomes reported
Other bias	Low risk	None noted

Sakoulas

**Methods**	Prospective randomized open label
**Participants**	Hospitalized hypoxic subjects with COVID-19 pneumonia
**Interventions**	Pts were randomized 1:1 to receive standard of care plus IV immunoglobulin 0.5g/kg/d with methylprednisolone 40mg 30 minutes before infusion for 3 days versus standard of care alone.Sixteen subjects received IV immunoglobulin and 17 standard of care
**Outcomes**	composite ventilation endpoint, death, or discharged from the hospital. rates of receipt of mechanical ventilation
**Notes**	Pts with moderate-to-severe hypoxia (sPo2 ≤ 96% on ≥ 4L O2 by nasal cannula) but not on mechanical ventilation were considered

Risk of bias table

Bias	Authors' judgement	Support for judgement
Random sequence generation (selection bias)	Low risk	a web-based computer-generated randomization procedure.
Allocation concealment (selection bias)	Low risk	When the randomization list was generated, M.G. placed the codes into individual sealed and sequentially numbered envelopes. The batch of sealed envelopes was stacked in sequential order and retained in a locked drawer in the Investigational Research Pharmacy.
Blinding of participants and personnel (performance bias)	High risk	open label
Blinding of outcome assessment (detection bias)	Unclear risk	no information provided
Incomplete outcome data (attrition bias)	Low risk	Immediately after randomization and notification of the principal investigator, one subject was immediately deemed unevaluable by the principal investigator and excluded due to a high risk of bacterial superinfection (
Selective reporting (reporting bias)	Low risk	all outcomes reproted
Other bias	Low risk	none detected

Tabarsi

**Methods**	randomized controlled trial
**Participants**	84 patients with severe COVID-19 were included: 52 in the IVIg group and 32 in the control group.
**Interventions**	The intervention group received IVIg at a dose of 400 mg/kg, IV, daily for three days. Both groups received hydroxychloroquine, lopinavir/ritonavir and supportive care.

**Outcomes**	mortality rate, the need for mechanical ventilation, length of stay in hospital and in Intensive Care Unit (ICU), and imaging findings were recorded and compared
**Notes**	Severe pneumonia cases were determined based on World Health Organization (WHO) case definitions for COVID-19 consisting of the following: respiratory rates: ≥ 30 breaths/min, $SpO_2 \leq 93\%$, and $PaO_2/FiO_2 \leq 300$ mmHg.

Risk of bias table

**Bias**	**Authors' judgement**	**Support for judgement**
Random sequence generation (selection bias)	Low risk	Block randomization method was used for randomization. Eight blocks, including ten patients, were generated by the Online Randomizer website
Allocation concealment (selection bias)	Unclear risk	no information provided
Blinding of participants and personnel (performance bias)	High risk	open label
Blinding of outcome assessment (detection bias)	Unclear risk	no information provided
Incomplete outcome data (attrition bias)	Low risk	no risk of attrition bias detected
Selective reporting (reporting bias)	Low risk	all outcomes reported
Other bias	Low risk	None noted

MAIN CHARACTERISTICS OF NON-RANDOMIZED STUDIES, INCLUDING RISK OF BIAS (ROBIN-1).

Characteristics of included studies

Cao

**Methods**	A multi-center retrospective study. The selected patients were enrolled and divided into two groups according to their treatment history: the IVIg group (high-dose IVIg therapy coupled with standard care following admission) and the control group (standard care only)
**Participants**	Pts with severe COVID-19. 26 patients who received high-dose IVIg with standard therapy and 89 patients who received standard therapy only were enrolled in this study
**Interventions**	IVIg administered within two weeks of disease onset at a total dose of 2 g/kg body weight, in addition to standard care.
**Outcomes**	The primary endpoint was 28-day mortality.
**Notes**	Efficacy of high-dose IVIg was assessed by using the Cox proportional hazards regression model and the Kaplan-Meier curve adjusted by inverse probability of treatment weighting (IPTW) analysis, and IPTW after multiple imputation (MI) analysis.

Risk of bias table

Bias	Authors' judgement	Support for judgement
Confounding	Unclear risk	Most of the baseline characteristics were balanced between the IVIg and control groups after adjustment. Nonetheless, imbalances might be inadequately controlled for in any retrospective analysis, even if multiple regression models were used to control for confounding factors.
Selection bias	Unclear risk	The selected patients were enrolled and divided into two groups according to their treatment history. The number of pts in the treatment group is substantially smaller than in control group.
Bias in measurement classification of interventions	Unclear risk	retrospective evaluation of pts in both treated and control group
Bias due to missing data	Low risk	small differences observed in the accuracy or completeness of the data retrieved in the 2 groups.
Bias in measurements of outcomes	Low risk	methods of outcome assessment were comparable across intervention groups.
Bias in selection of the reported results	Low risk	all the outcomes reported in treated and controls

Esen

**Methods**	retrospective cohort study
**Participants**	COVID-19 pts with severe disease. Patients had received preliminary standard intensive care (SIC) according to a local treatment algorithm, either alone or along with IVIG 5% at 30 g/day for 5 days.
**Interventions**	IVIG or ST. Out of 93 patients, 51 had received IVIG and 42 had not.. Standard pharmaceutical treatment comprised hydroxychloroquine , favipiravir, azithromycin, oseltamivir , tocilizumab or anakinra depending on inflammatory markers, methylprednisolone (200 mg/day), high dose vasopressors in case of septic shock and vitamin C (6 g/day i.v. for 7 days). To this treatment regimen IVIG 5% was added at a dose 30 g/day for five consecutive days, on an individual case basis in one of the two ICUs. F
**Outcomes**	mortality; changes in biomarkers
**Notes**	

Risk of bias table

**Bias**	**Authors' judgement**	**Support for judgement**
Confounding	High risk	the non-IVIG-treated group appeared to suffer greater disease severity at baseline.. The IVIG group was younger and had slightly lower baseline disease scores though. There were no major differences in concomitant COVID-19 treatments while distribution of concurrent diseases was different with 1.5 to 2-fold higher prevalence of diabetes and malignancies in the IVIG group and of chronic cardiac, chronic renal and cerebrovascular disease in the SIC group . Accordingly, proBNP and troponin levels at baseline were lower in the IVIG group,
Selection bias	High risk	high-dose IVIG was added to SIC in one of two separate wards (in the Department of Internal Medicine) without informing the other (in the Department of Anesthesiology and Intensive Care).Although assignment to either of the two wards was made by an independent gatekeeper and principally driven by free capacities, it might have been biased in case of simultaneous admissions by the better logistics for extracorporeal membrane oxygenation (ECMO) in the ward with eventually more severe patients.
Bias in measurement classification of interventions	Unclear risk	retrospective evaluation of pts in both treated and control group
Bias due to missing data	Low risk	no differences observed in the accuracy or completeness of the data retrieved in the 2 groups.
Bias in measurements of outcomes	Low risk	methods of outcome assessment were comparable across intervention groups.

Bias in selection of the reported results	Low risk	all the outcomes reported in treated and controls

Hou

**Methods**	a single-center retrospective cohort study in China
**Participants**	113 adult patients with laboratory-confrmed severe COVID-19
**Interventions**	IVIG vs ST
**Outcomes**	The primary outcome was the composite end point, including death and the use of mechanical ventilation. The secondary outcome was the length of hospital stay.
**Notes**	

Risk of bias table

**Bias**	**Authors' judgement**	**Support for judgement**
Confounding	High risk	Compared with patients who did not receive IVIG, fewer patients who received IVIG therapy had coronary heart disease [0 (0) vs 7 (10.6%), P=0.021]. In addition, patients who received IVIG therapy had a higher body temperature [38.9 (38.2– 39.0) vs 38.0 (37.5–38.8), P=0.002] before hospital admission, a higher white blood cell count [7.45 (4.73– 9.42) vs 5.00 (3.68–6.79), P
Selection bias	Low risk	The control group and intervention included patients hospitalized during the same time period and with similar disease characteristics at the time of admission
Bias in measurement classification of interventions	Unclear risk	retrospective evaluation of pts in both treated and control group
Bias due to missing data	Low risk	no differences observed in the accuracy or completeness of the data retrieved in the 2 groups.
Bias in measurements of outcomes	Low risk	methods of outcome assessment were comparable across intervention groups.
Bias in selection of the reported results	Low risk	all the outcomes reported in treated and controls

Huang

**Methods**	A retrospective cohort study based on propensity score matching
**Participants**	non-severe covid-19 pts
**Interventions**	IVIG vs controls

**Outcomes**	Primary outcomes included the severity and mortality rates. Secondary outcomes included the duration of fever, virus clearance time, length of hospital stay, and use of antibiotics.
**Notes**	Such imbalances might be inadequately controlled for in any retrospective analysis, even if multiple regression models were used to control for confounding factors.

Risk of bias table

**Bias**	**Authors' judgement**	**Support for judgement**
Confounding	High risk	patients who were older (56 vs 36 years, p <0.001), more commonly had a comorbidity (51.1% vs 18.7%, p<0.01), were more likely to be treated with IVIG. Moreover, corticosteroids (20% vs 7.4%, p = 0.003), thymosin α (88.9% vs 24.2%, p< 0.001)and lopinavir/ ritonavir (46.7% vs 15.5%, p< 0.001) were more frequently used in the IVIG. As there were significant differences in baseline characteristics, patients were selected by PSM method according to a 1:2 ratio.
Selection bias	High risk	In this study, 45 patients received IVIG therapy and 594 patients received standard therapy. The study was a retrospective research, and the dose and duration of IVIG was not randomized. As almost all severe cases in our hospital received IVIG treatment, no control group could be used to evaluate the efficacy of IVIG in severe cases. As a tentative therapy, and considering the side effects and high price, IVIG was used selectively for non-severe patients with more risk factors for a worse evolution of COVID-19 according to the joint discussions of at least five experts from the Shanghai Medical Expert Group for the
Bias in measurement classification of interventions	Unclear risk	The records of 664 patients with COVID-19 admitted to Shanghai Public Health Clinical Center between January 20, 2020 and June 10, 2020 were reviewed retrospectively
Bias due to missing data	Low risk	no differences observed in the accuracy or completeness of the data retrieved in the 2 groups.
Bias in measurements of outcomes	Low risk	methods of outcome assessment were comparable across intervention groups.
Bias in selection of the reported results	Low risk	all the outcomes reported in treated and controls

Liu

**Methods**	Retrospective cohort study. Each patient treated with IVIG was matched with one untreated patient. Logistic regression and inverse probability weighting (IPW) were used to control confounding factors.
**Participants**	Pts with severe covid-19. The study included 850 patients (421 IVIG-treated patients and 429 non-IVIG-treated patients). After matching, 406 patients per group remained
**Interventions**	IVIG vs ST
**Outcomes**	The primary outcome was defined as 28-day all-cause mortality after propensity matching analysis.The secondary outcomes were defined as ARDS, DIC, myocardial injury, acute hepatic injury, shock, acute kidney injury (AKI), non-invasive mechanical ventilation, invasive mechanical ventilation, prone position ventilation, continuous renal replacement therapy and ECMO between the two groups (treated versus untreated patients)
**Notes**	

Risk of bias table

**Bias**	**Authors' judgement**	**Support for judgement**
Confounding	Unclear risk	Most of the baseline characteristics were balanced between the IVIg and control groups after adjustment. Nonetheless, imbalances might be inadequately controlled for in any retrospective analysis, even if multiple regression models were used to control for confounding factors
Selection bias	Low risk	In order to avoid immortal time bias, we matched each patient treated with IVIG with an untreated patient, according to the day of admission for treatment (or lack of treatment). In case of multiple matching patients, one untreated matching patient was thus ra
Bias in measurement classification of interventions	Unclear risk	retrospective evaluation of pts in both treated and control group
Bias due to missing data	Low risk	no differences observed in the accuracy or completeness of the data retrieved in the 2 groups.
Bias in measurements of outcomes	Low risk	methods of outcome assessment were comparable across intervention groups.
Bias in selection of the reported results	Low risk	all the outcomes reported in treated and controls

Shao

**Methods**	a multicenter retrospective cohort study
**Participants**	325 patients with laboratory-confirmed critical COVID-19 were enrolled from 4 government-designated COVID-19 treatment centres in southern China from December 2019 to March 2020.
**Interventions**	174 cases used IVIG and 151 cases did not.
**Outcomes**	The primary outcomes were 28- and 60-day mortality, and the secondary outcomes were the total length of in-hospital and the total duration of the disease
**Notes**	Subgroup analysis was carried out according to clinical classification of COVID-19, IVIG dosage and timing

Risk of bias table

**Bias**	**Authors' judgement**	**Support for judgement**
Confounding	High risk	Comparisons of baseline characteristics between the two groups showed that the disease was more severe in the IVIG group (older age, higher APACH II scores and SOFA scores, higher levels of total bilirubin, direct bilirubin, creatinine, Creactive protein, IL-6 and lactate, but lower platelets and lymphocyte count. To adjust for confounders, the Cox proportional hazards model was used, but the possibility of unrecognized or unmeasured confounding variables. cannot be ruled out
Selection bias	High risk	the authors retrospectively collected the clinical and outcome data of critical COVID-19 patients, including both severe type and critical type, from 4 government-designated treatment centres in three cities. The dose and timing of IVIG administration in each centre may not be exactly consistent.
Bias in measurement classification of interventions	Unclear risk	retrospective evaluation of pts in both treated and control group
Bias due to missing data	Low risk	no differences observed in the accuracy or completeness of the data retrieved in the 2 groups.
Bias in measurements of outcomes	Low risk	methods of outcome assessment were comparable across intervention groups.
Bias in selection of the reported results	Low risk	all the outcomes reported in treated and controls
